# Identification and Validation of Quantitative Trait Loci (QTL) for Canine Hip Dysplasia (CHD) in German Shepherd Dogs

**DOI:** 10.1371/journal.pone.0096618

**Published:** 2014-05-06

**Authors:** Lena Fels, Ottmar Distl

**Affiliations:** Institute for Animal Breeding and Genetics, University of Veterinary Medicine Hannover, Hannover, Germany; CSIRO, Australia

## Abstract

Canine hip dysplasia (CHD) is the most common hereditary skeletal disorder in dogs. To identify common alleles associated with CHD, we genotyped 96 German Shepherd Dogs affected by mild, moderate and severe CHD and 96 breed, sex, age and birth year matched controls using the Affymetrix canine high density SNP chip. A mixed linear model analysis identified five SNPs associated with CHD scores on dog chromosomes (CFA) 19, 24, 26 and 34. These five SNPs were validated in a by sex, age, birth year and coancestry stratified sample of 843 German Shepherd Dogs including 277 unaffected dogs and 566 CHD-affected dogs. Mean coancestry coefficients among and within cases and controls were <0.1%. Genotype effects of these SNPs explained 20–32% of the phenotypic variance of CHD in German Shepherd Dogs employed for validation. Genome-wide significance in the validation data set could be shown for each one CHD-associated SNP on CFA24, 26 and 34. These SNPs are located within or in close proximity of genes involved in bone formation and related through a joint network. The present study validated positional candidate genes within two previously known quantitative trait loci (QTL) and a novel QTL for CHD in German Shepherd Dogs.

## Introduction

Canine hip dysplasia (CHD) is a common trait in most dog breeds. This orthopedic condition causes instability and subluxation of the hip with secondary signs of osteoarthritis and clinical signs of lameness. Breed prevalences vary widely from 1% to 75% [1]. In German Shepherd Dogs, prevalence of CHD is estimated at 35% [2]. There is strong evidence in support of a genetic predisposition to CHD in German Shepherd Dogs and many other dog breeds. Heritability estimates for German Shepherd Dogs from different European countries were at h^2^  =  0.20 to 0.35 [2]–[4]. Results from radiographic screenings for CHD of 48,367 German Shepherd Dogs born in 2001–2007 were used for estimation of heritabilities in threshold and mixed linear-threshold models [4]. In this sample of German Shepherd Dogs, heritability was 0.25 for CHD in the linear model and 0.19–0.27 for binary trait definitions regarding only borderline as CHD-affected and mild to severe CHD cases as CHD-affected versus CHD-free dogs. In a study of 13,124 Australian German Shepherd Dogs born between 1976 and 2005, the heritability of the summed phenotype constructed from nine ordinally-scored British Veterinary Association Hip Traits was 0.30 [5]. Linear models are commonly applied for genetic analyses of CHD as this methodology most correctly reflects the underlying nature of the data [4], [5].

Complex segregation analyses demonstrated involvement of a major gene for the German Shepherd Dog [6], [7]. Genome-wide linkage studies showed nine genome-wide significant quantitative trait loci (QTL) for CHD in German Shepherd Dogs [8]. A linkage study in a Labrador Retriever-Greyhound crossbred family revealed twelve dog chromosomes (CFA) with chromosome-wide significant markers for CHD [9]. In Portuguese Water Dogs, QTL for signs of CHD were demonstrated on CFA1 and 3 [10], [11]. A genome-wide association study (GWAS) for CHD and osteoarthritis (OA) across several dog breeds including Labrador Retriever-Greyhound crosses identified four CHD-associated and two OA-associated SNPs. The CHD-associated SNPs were located on CFA3, 11 and 30 [12], but not within QTL of the Labrador Retriever-Greyhound crossbred linkage study [8]. In 174 Bernese Mountain Dogs, two different CHD-regions were identified on CFA14. A third CHD-associated region was located on CFA37 [13]. A Dutch study on 48 CHD-affected and 30 CHD-free Labrador Retrievers revealed significant SNPs on CFA8 [14] within a previously reported quantitative trait locus (QTL) in German Shepherd Dogs [8]. A 10-bp intronic deletion haplotype within *FBN2* on CFA11 was shown to be associated with CHD [15]. Dogs homozygous for this haplotype had significantly less *FBN2* mRNA in their femoral head articular cartilage [15]. The mutant *FBN2* haplotype was identified in 49 different breeds, but homozygous mutant haplotypes were only prevalent in Labrador and Golden Retrievers [16].

The objective of the present study was to perform a GWAS on 192 dogs to identify SNPs associated with CHD, followed by a validation of CHD-associated SNPs in a stratified sample of 843 dogs. We have chosen the German population of German Shepherd Dogs as this population is well suited for an association study, since this breed represents one of the largest purebred dog populations in Europe with a large phenotypic and genetic variance for CHD and a consistent recording system of CHD including the collection of EDTA-blood samples.

## Results

**Figure 1:**
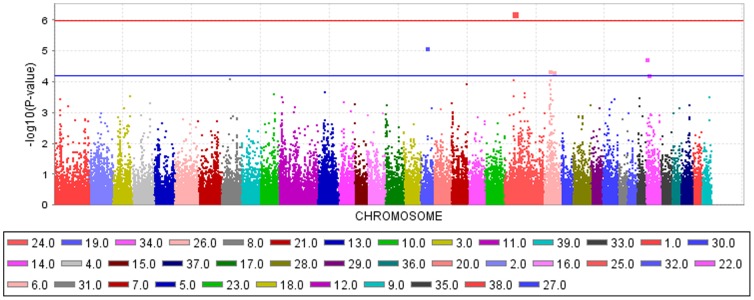
Manhattan plot of –log_10_P-values of the genome-wide association study for the canine hip dysplasia score in German Shepherd Dogs using a mixed linear model analysis. On the X-axis, the SNPs are given by dog chromosome number. The –log_10_P-values for each SNP genotype effect are plotted against the SNP position on each chromosome. Chromosomes are differentiated by colors. The color keys are given below the plot. The red line indicates a –log_10_P-value of 4.3 and the blue line indicates the threshold of the –log_10_P-values for genome-wide significance after correcting for multiple testing.

The genome-wide scan using the canine 127K Affymetrix SNP chip (Affymetrix, Santa Clara, CA, USA) revealed five SNPs with –log_10_P-values>4.3 suggestive for association with CHD (Figure 1). We detected a genome-wide significant CHD-associated SNP on CFA24 in a region which was not significant in a previous linkage study for German Shepherd Dogs (Table S1) [8]. We determined the genome-wide threshold for significance at a -log_10_P-value >5.98 which corresponds to a P-value <0.05 after applying the Bonferroni correction for multiple testing. The quantile-quantile (Q-Q) plots illustrated that inflation due to stratification effects had been removed by the mixed linear model employed for CHD (Figure S1).

The validation study included the five most significant SNPs on CFA19, 24, 26 and 34. Two SNPs were located on CFA34 and each one SNP on CFA19, 24 and 26 (Table S2). All these five SNPs were evaluated in 843 German Shepherd Dogs. The minor allele frequencies (MAF) ranged from 0.07 to 0.48 (Table 1). Each one SNP on CFA24, 26 and 34 was significantly associated with CHD in the validation set, whereas the SNP on CFA19 and one SNP on CFA34 did not pass validation (Table 2). The CHD-QTL on CFA24, 26 and 34 were confirmed in the analyses when dogs with the CHD grades C and D were separately compared with all CHD-free dogs (Tables 3–4). The CHD-QTL on CFA24 was also significant for the separate analysis for CHD-E (Table 5). A combined analysis for dogs free from CHD and dogs with CHD-D and CHD-E gave slightly higher –log_10_P-values compared to the analysis with CHD-A versus CHD-D dogs (data not shown).

**Table 1:** SNP-IDs of the five SNPs genotyped in the validation set of 843 German Shepherd Dogs, their position on dog chromosome (CFA), their chromosomal position according to CanFam2.0, their minor alleles, their minor allele frequencies (MAF) in all dogs and among 277 unaffected dogs (CHD-grade A) and 566 affected dogs with 349 dogs classified as CHD-grade C, 152 dogs as CHD-grade D and 65 dogs as CHD-grade E.

SNP-ID	CFA	Position	Minor	MAF					
(CanFam2.0)		(Mb)	allele	Total	CHD-A	CHD-C/D/E	CHD-C	CHD-D	CHD-E
TIGRP2P265674	19	35.533	C	0.067	0.070	0.065	0.064	0.060	0.079
BICF2S2367279	24	28.944	G	0.483	0.247	0.607	0.419	0.655	0.587
BICF2P281364	26	17.181	G	0.366	0.506	0.299	0.293	0.305	0.318
BICF2P1086886	34	4.239	T	0.300	0.208	0.344	0.344	0.333	0.365
BICF2P355865	34	39.346	A	0.322	0.287	0.334	0.343	0.332	0.341

**Table 2:** Odds ratios (OR) of the five SNPs genotyped with their 95% lower (CL) and upper confidence limits (CU), χ^2^- and -log_10_P-values for genotypic, allelic and trend association considering 277 CHD-A German Shepherd Dogs as controls and 566 German Shepherd Dogs classified as CHD-C/D/E as cases.

SNP-ID	CFA	OR	OR-CL	OR-CU	Genotype		Allele		Trend	
					χ^2^	-log_10_P-value	χ^2^	-log_10_P-value	χ^2^	-log_10_P-value
TIGRP2P265674	19	1.01	0.73	1.65	2.96	0.64	0.19	0.18	0.19	0.18
BICF2S2367279	24	4.70	3.72	5.94	130.75	28.39	178.66	40.02	115.71	26.26
BICF2P281364	26	0.42	0.34	0.52	62.04	13.47	65.47	15.22	56.71	13.30
BICF2P1086886	34	1.99	1.55	2.55	29.39	6.38	30.39	7.45	27.39	6.78
BICF2P355865	34	0.78	0.62	0.98	4.65	1.01	4.51	1.47	4.60	1.50

**Table 3:** Odds ratios (OR) of the five SNPs genotyped with their 95% lower (CL) and upper confidence limits (CU), χ^2^-values and -log_10_P-values for genotypic, allelic and trend association considering 277 CHD-A German Shepherd Dogs as controls and 349 German Shepherd Dogs classified as CHD-C as cases.

SNP-ID	CFA	OR	OR-CL	OR-CU	Genotype		Allele		Trend	
					χ^2^	-log_10_P-value	χ^2^	-log_10_P-value	χ^2^	-log_10_P-value
TIGRP2P265674	19	1.11	0.70	1.75	3.57	0.77	0.20	0.19	0.20	0.18
BICF2S2367279	24	4.36	3.37	5.63	105.50	22.91	133.23	30.09	88.47	20.29
BICF2P281364	26	0.41	0.32	0.51	56.35	12.24	56.53	13.26	49.13	11.62
BICF2P1086886	34	1.99	1.53	2.60	26.24	5.70	26.17	6.50	24.24	6.07
BICF2P355865	34	0.77	0.60	0.99	4.51	0.98	4.22	1.40	4.34	1.43

**Table 4:** Odds ratios (OR) of the five SNPs genotyped with their 95% lower (CL) and upper confidence limits (CU), χ^2^- and -log_10_P-values for genotypic, allelic and trend association considering 277 CHD-A German Shepherd Dogs as controls and 152 German Shepherd Dogs classified as CHD-D as cases.

SNP-ID	CFA	OR	OR-CL	OR-CU	Genotype		Allele		Trend	
					χ^2^	-log_10_P-value	χ^2^	-log_10_P-value	χ^2^	-log_10_P-value
TIGRP2P265674	19	1.18	0.66	2.11	2.66	0.58	0.30	0.23	0.31	0.24
BICF2S2367279	24	5.78	4.21	7.93	106.83	23.20	127.20	28.77	87.40	20.05
BICF2P281364	26	0.43	0.32	0.58	32.68	7.10	31.09	7.61	28.64	7.06
BICF2P1086886	34	1.90	1.38	2.63	21.27	4.62	15.47	4.08	14.18	3.78
BICF2P355865	34	0.81	0.60	1.10	1.93	0.42	1.83	0.76	1.83	0.75

**Table 5:** Odds ratios (OR) of the five SNPs genotyped with their 95% lower (CL) and upper confidence limits (CU), χ^2^- and -log_10_P-values for genotypic, allelic and trend association considering 277 CHD-A German Shepherd Dogs as controls and 65 German Shepherd Dogs classified as CHD-E as cases.

SNP-ID	CFA	OR	OR-CL	OR-CU	Genotype		Allele		Trend	
					χ^2^	-log_10_P-value	χ^2^	-log_10_P-value	χ^2^	-log_10_P-value
TIGRP2P265674	19	0.88	0.42	1.82	0.13	0.15	0.12	0.14	0.13	0.15
BICF2S2367279	24	4.33	2.89	6.51	53.32	11.58	54.71	12.85	41.15	9.85
BICF2P281364	26	0.45	0.30	0.69	15.95	3.46	14.46	3.84	13.92	3.72
BICF2P1086886	34	2.19	1.44	3.33	15.51	3.37	13.70	3.84	13.76	3.68
BICF2P355865	34	0.78	0.51	1.18	1.45	0.31	1.43	0.63	1.45	0.64

The strongest association was found for the SNP on CFA24. In all analyses considering CHD-free German Shepherd Dogs versus mildly to severely CHD-affected dogs (C+D+E or C or D or E or D+E), BICF2S2367279 reached -log_10_P-values at 10–40. Odds ratios (ORs) were highest for BICF2S2367279 with values at 4.3–5.8. The CHD-associated genotype G/G of BICF2S2367279 on CFA24 occurred in 312/843 dogs and 27/312 were free from CHD and all other 285/312 were CHD-affected. Corresponding figures for BICF2S2367279 on CFA26 were for the CHD-associated genotype A/A were 369/843, 66/369 (CHD-free) and 303/369 (CHD-affected), respectively. The joint genotypes G/G and A/A had a frequency of 222/843 and only 10/222 were free from CHD, whereas 212/222 were CHD-affected.

The proportion of phenotypic variance of CHD explained by the cumulative effects of all five SNPs was 19.6% (all CHD-grades), 21.6% (CHD-A vs C), 31.6% (CHD-A vs CHD-D), 20.7% (CHD-A vs. CHD-E) and 28.0% (CHD-A vs. CHD-D and CHD-E).

## Discussion

The present GWAS identified a novel genome-wide significant CHD-QTL on CFA24, which was validated in a large sample of German Shepherd Dogs stratified by sex, age, birth year, CHD-status and ancestry. A slight overestimation of the –log_10_P-values may be assumed in the detection sample because we used a linear model and not a cumulative logit model. However, the –log_10_P-value for the SNP on CFA24 was still below the Bonferroni significance threshold of 0.05 (Table S3). Further two QTL from a previous linkage study [8] could be confirmed through a GWAS and a validation study.

In close proximity to the SNP BICF2S2367279 on CFA24, the gene *SRC* (*v-src sarcoma (Schmidt-Ruppin A-2) viral oncogene homolog*) is located. This gene is involved in bone formation mediating cytoskeletal reorganization as well as osteoclast survival in response to RANKL in vitro [17]. Targeted disruption of *src* function in mice caused osteopetrosis, which is characterized by increased bone density due to impairment of osteoclast bone resorbing activity. Osteoclasts from src-null mice failed to form ruffled borders impairing their ability to resorb bone matrix [18].

On CFA26, the SNP BICF2P281364 is located within *KSR2* (*kinase suppressor of ras 2*) which encodes a scaffold protein interacting with members of the RAS/MAPK signalling pathway [19]. KSR2 has been suggested to be involved in activation of molecules like p38 MAPK, cell cycle control proteins and proteins of the ubiquitin proteasome. The p38α and p38β MAPK proteins are important regulators of bone homeostasis controlling expression and activation of transcription factors implicated in osteoblastogenesis like RUNX2 [20], [21]. In addition, KSR2 is predicted to mediate PI3K activation. Via PI3K, a RANKL mediated signalling pathway is activated which negatively regulates osteoclast survival [22], [23].

On CFA34, the SNP BICF2P1086886 is located approximately 0.7 Mb downstream of the *triple functional domain (PTPRF interacting)* (*TRIO*) gene. The protein encoded by *TRIO* contains three functional domains, a serine/threonine kinase domain and two guanine nucleotide exchange factor (GEF) domains for the family of Rho-like GTPases, specific for Rac1 and RhoA [24]. Rac1 and RhoA act as antagonists, both playing an important role in chondrogenic proliferation and differentiation [25], [26]. Chondrocyte-specific deletion of Rac1 in mice leads to dwarfism due to reduced chondrocyte proliferation [27]. Inhibition of Rac1 expression in micromass culture resulted in reduced mRNA levels of the chondrogenic markers collagen II and aggrecan, and decreased accumulation of glycosaminoglycans indicating that Rac1 promotes chondrogenesis [28]. Rac1-deficient chondrocytes had severely reduced levels of inducible nitric oxide synthase protein (iNOS) and nitric oxide production [29]. Mice deficient for iNOS had reduced chondrocyte proliferation and resembled the phenotype of Rac1-deficient growth plates. RhoA overexpression in chondrogenic ATDC5 cells resulted in increased proliferation and a marked delay of hypertrophic differentiation, whereas inhibition of Rho/ROCK signaling inhibited chondrocyte proliferation and accelerates hypertrophic differentiation [30]. Therefore, changing the balance between the GTPases RhoA and Rac1 due to mutations in *trio* will lead to disturbances in cartilage development.

In agreement with previous reports, the CHD-associated regions and the candidate genes identified here, support the key role of enchondral bone formation in the pathogenesis of CHD [8]–[16]. A candidate region identified in German Shepherd Dogs [8] and Labrador Retrievers [14] on CFA8 at 29 Mb harbours the potential candidate gene *LRR1*. The encoded protein is involved in proteoglycan synthesis through NF-κB signalling transduction and cartilage integrity like iNOS and MAPK proteins [31]. In Bernese Mountain Dogs, *FN1* was identified as a candidate gene for CHD [13]. FN1 had been proposed to have a function in matrix organization of cartilage [32]. In an across-breed GWAS, *EVC* and *EVC2* on CFA3, *PTPRD* on CFA11 and *MFAP1* on CFA30 were suggested as CHD candidate genes [12]. All these genes are highly expressed in cartilage and mutations in these genes can cause chondrodysplasia (*EVC*, *EVC2*), Marfan syndrome (*MFAP1*) or restless legs syndrome (*PTPRD*) [12].

For the candidate genes on CFA24, 26 and 34, we could find a joint network involved in bone formation using GENEMANIA (http://www.genemania.org/). Joint co-expression and physical interactions had been indicated for *TRIO, SRC* and *KSR2*. This may indicate the QTL identified harbour genes contributing to CHD on the same pathways. Using the candidate genes *LRR1*, *FN1*, *PTPRD* and *MFAP1*, we could extend the network for *TRIO, SRC* and *KSR2*, primarily through *RAC1*.

In conclusion, this study identified and corroborated CHD-loci on three different chromosomes for German Shepherd Dogs and is a further step towards elucidation of the genes underlying CHD. The three validated and significantly CHD-associated SNPs are located within or in close proximity to genes which are involved in a joint network regulating bone formation, osteoclast activity, chondrocyte proliferation and differentiation.

## Material and Methods

### Ethics Statement

All animal work has been conducted according to the national and international guidelines for animal welfare. The dogs in this study were included with the consent of their owners. The blood samples were collected by veterinarians during the routine examination for CHD regarding principles of good veterinary practice. These diagnostic procedures had to be carried out anyway. All blood-sampling of dogs was done in veterinary practices for small animals by trained staff. CHD examinations are compulsory for all dogs intended for breeding and these examinations were performed in veterinary clinics and practices according to the rules for hip screening of the Fédération Cynologique Internationale (FCI). Approval from the ethics committee was not obtained because blood sampling was done during a diagnostic veterinary procedure according to the German Animal Welfare Law (released on 05/18/2006, last changes on 08/07/2013).

### Animals

A total of 1035 German Shepherd Dogs had been included in the present association study for CHD. The sample for the GWAS to identify significantly associated SNPs contained 192 dogs and the validation set 834 dogs. First, we selected the dogs for the GWAS and then, the validation sample was collected from available databases and bio-banks. The study population was a stratified sample from all registered and X-rayed dogs of the German population of German Shepherd Dogs born between 2000 and 2005. Dogs had an age at examination of 12–14 months in each cohort. Coancestry among cases and controls as well as within cases and within controls was minimized. In addition, the samples used were stratified by sex, age at radiographic examination, birth year, coancestry and CHD-affection. The pedigree records from eleven generations were employed to calculate relationship coefficients among all dogs using PEDIG software [33]. The study population had mean relationship coefficients among all individual dogs <0.1%. Cases and controls were chosen with equal proportions of sexes and birth cohorts from the years 2000–2005. Dogs were 12–14 months old at radiographic examination. Radiographs and CHD-scores according to the official guidelines of the FCI as well as EDTA-blood samples for parentage testing were collected by the Association for German Shepherd Dogs (SV, Augsburg). Parentage testing is mandatory for all German Shepherd Dogs intended for breeding. For all dogs included in the present study, parentage has been confirmed using an approved set of microsatellites for parentage control. The radiographs were made by veterinarians officially approved by the association of radiographic diagnosis for genetically influenced skeletal diseases of small animals (GRSK) and the German Association for Dog Breeding and Husbandry (VDH) strictly following the requirements for radiographs of hip joints of the FCI. All X-rays were evaluated by a recognized veterinary expert and a subsample of 200 X-rays was re-evaluated by further two experts. Consistency of diagnoses was >99%. These officially recorded CHD-grades were supplied by the SV and used in all subsequent analyses. CHD-A includes dogs with normal hips, CHD-C dogs with slight signs of CHD, CHD-D dogs with moderate signs of CHD and CHD-E dogs with severe signs of CHD using X-rays from both hip joints. Dogs with mild, moderate or severe signs of CHD were defined as CHD-affected. Controls were dogs free from any signs of CHD.

### Genome-wide Association Study

A genome-wide screening for polymorphic and CHD-associated SNPs was performed using the canine 127K Affymetrix SNP chip (Affymetrix). This sample included 192 German Shepherd Dogs whereof 96 were free from CHD, 65 mildly, 22 moderately and 9 severely CHD-affected. Cases and controls were balanced by CHD score, sex, birth year, age at veterinary radiographic examination and coancestry. The animals were unrelated at least at the grandparent level. Relationship coefficients with the validation sample were as low as possible and did not exceed 0.1%, on average. The dogs from both data sets, the detection and validation samples, were unrelated at least on the grandparent level. Quality criteria were minor allele frequency (MAF) >0.05, genotyping rate per SNP and animal >0.90 and HWE (p<0.00001). After filtering for quality criteria, 47,729 SNPs remained for the final analysis. Data analysis was done for the CHD-score as quasi-continuous target trait. We used a mixed linear model with sex and genotype as fixed effects and a random animal effect through an identity-by-state-kinship (IBS) matrix. A Q-matrix to detect population structure was estimated using principal components (PCAs). We also tested several extended models employing up to five PCAs to show the robustness of the outcome of the GWAS. All these models yielded the same highly significant associated SNP as the final model. Thus, adding principal components for cryptic data structure did not change the results of the final model. Thus, the IBS matrix reflected the genomic relationship matrix among all individuals genotyped and captured the relatedness among animals as well as the cryptic family structure. The analysis was run using TASSEL, version 3.0.164 [34].

### Validation Study

Cases and controls were matched by sex, birth year, age at radiographic examination, CHD score and coancestry (Table 6). We edited 843 German Shepherd Dogs out of a group of 12,096 dogs which fitted our study design and had mean relationship coefficients <0.1% with any other individual dog in the sample. The animals were born in 2000–2005 and purebred following the rules of the SV. Distribution of CHD-scores in the 405 males and the 438 females was as follows. In the males, 136 (16.1%) CHD-A, 176 (20.9%) CHD-C, 72 (8.5%) CHD-D and 21 (2.5%) CHD-E; in the females, 141 (16.7%) CHD-A, 173 (20.5%) CHD-C, 80 (9.5%) CHD-D and 44 (5.2%) CHD-E. Dogs scored as CHD-A were treated as unaffected (n = 277), dogs scored as CHD-C (n = 349), CHD-D (n = 152) and CHD-E (n = 65) as affected. Association analyses were performed using odds ratios with their 95% confidence limits and χ^2^-tests for genotypic and allelic associations and the allelic trend with the CHD status (CHD-A vs. CHD-C/D/E) and with CHD-A versus CHD-C, CHD-D or CHD-E. We tested for the different CHD grades separately to see whether the association is consistent across the different CHD-grades or may be caused by a specific CHD-grade. The CASECONTROL procedure of SAS/Genetics, version 9.3 (Statistical Analysis System, Cary, NC, USA, 2013), was used for these calculations.

**Table 6:** Number of German Shepherd Dogs included in the validation study. Distribution of the 843 dogs by canine hip dysplasia (CHD) score, sex and birth year is given.

Birth year	CHD-A		CHD-C		CHD-D		CHD-E		Total	
	male	female	male	female	male	female	male	female	male	female
2000/2001	35	34	63	58	16	21	10	24	124	137
2002	32	33	31	25	23	18	5	11	91	87
2003	27	27	29	32	14	19	4	4	74	82
2004/2005	42	47	43	58	19	22	2	5	116	132
	136	141	176	173	72	80	21	44	405	438
Total	277		349		152		65		843	

### DNA Preparation and Genotyping

DNA was isolated from EDTA-blood samples using the NucleoSpin Kit 96 Blood Quick Pure Kit (Macherey-Nagel, Düren, Germany). Five SNPs on CFA19, 24, 26 and 34 were chosen for validation from the GWAS. Genotyping of the validation SNP set consisting of five SNPs was performed using the ABI Prism SNaPshot™ Multiplex System (Life Technologies by Applied Biosystems, Darmstadt, Germany) (Table S2). Single base extension (SBE) primers were designed using online primer design software BatchPrimer3 (http://probes.pw.usda.gov/cgi-bin/batchprimer3/batchprimer3.cgi). According to the manufacturer's instructions, the primer sequences should differ in length by at least four to six nucleotides and should not contain possible extendable hairpin structures. All primers used here had undergone HPLC purification. The primer design was in this way that a difference of seven nucleotides between the SBE primers was achieved by adding non-homologous polynucleotides (poly (dNTP)) at the 5′end. The SBE detection was performed using an ABI Genetic Analyzer 3500 (Life Technologies by Applied Biosystems). Data evaluation was done using GeneMapper, version 4.2 (Life Technologies by Applied Biosystems).

### Statistical Analysis

Allele and genotype frequencies of the SNPs were calculated for the different CHD-grades as well as the observed heterozygosity (H_0_), the polymorphism information content (PIC) and Hardy-Weinberg equilibrium were estimated using the ALLELE procedure of SAS/Genetics. Association analyses were performed using odds ratios with their 95% confidence limits and χ^2^-tests of the CASECONTROL procedure of SAS/Genetics for genotypic and allelic associations and the allelic trend with the CHD status (CHD-A vs. CHD-C/D/E) and with CHD-A versus CHD-C, CHD-D or CHD-E. These four definitions of phenotypes were used for all analyses. A SNP was regarded as genome-wide significantly associated for –log_10_P-values>5.98 and suggestive for association with –log_10_P-values>4.3. The same cutoffs were applied for the validation study. A general linear model including all CHD-associated SNPs was used to estimate the proportion of the phenotypic variance explained by these SNPs. Calculations were performed using SAS, version 9.3.

## Supporting Information

Figure S1 **Q-Q-plot of expected –log_10_P-values versus observed–log_10_P-values from the mixed linear model analysis for canine hip dysplasia score in German Shepherd Dogs.** Shown are all 47,729 SNPs included in the genome-wide association analysis with the grey line corresponding to the null hypothesis of no association.
(DOC)

Table S1 Results of the genome-wide association study for canine hip dysplasia in 192 German Shepherd Dogs. Given are the SNP-IDs with their locations on the dog chromosome according to CanFam2.0, the minor allele frequencies (MAF) in the sample and –log_10_P-values (P-MLM) from the mixed linear model analysis. The SNP BICF2S2367279 exceeds the threshold (5.98) for genome-wide significance.
(DOC)

Table S2 Single nucleotide polymorphisms (SNPs) with their chromosomal positions on CanFam2.0, adjacent genes, SBE primers, SBE orientations and SNP motifs genotyped in the validation set including 843 German Shepherd Dogs.
(DOC)

Table S3 Comparison of -log_10_P-values using a cumulative logit and a linear model for an association analysis of canine hip dysplasia genotyped in the detection sample including 192 German Shepherd Dogs considering 96 CHD-A dogs as controls and 65 mildly, 22 moderately and 9 severely CHD-affected dogs. Shown are the raw -log_10_P-values of the five highest CHD-associated SNPs for a model with genotype as the sole effect and the overestimation of the raw -log_10_P-values through a linear model.
(DOC)
