# Supplementary material for: Identification and Validation of Quantitative Trait Loci (QTL) for Canine Hip Dysplasia (CHD) in German Shepherd Dogs
Source: PLoS One. 2014 May 6;9(5):e96618. doi: 10.1371/journal.pone.0096618 (PMC4011879; doi:10.1371/journal.pone.0096618)
Supplement: Figure S1 — Q-Q-plot of expected –log10P-values versus observed–log10P-values from the mixed linear model analysis for canine hip dysplasia score in German Shepherd Dogs. Shown are all 47,729 SNPs included in the genome-wide association analysis with the grey line corresponding to the null hypothesis of no association. (DOC) [file pone.0096618.s001.doc]

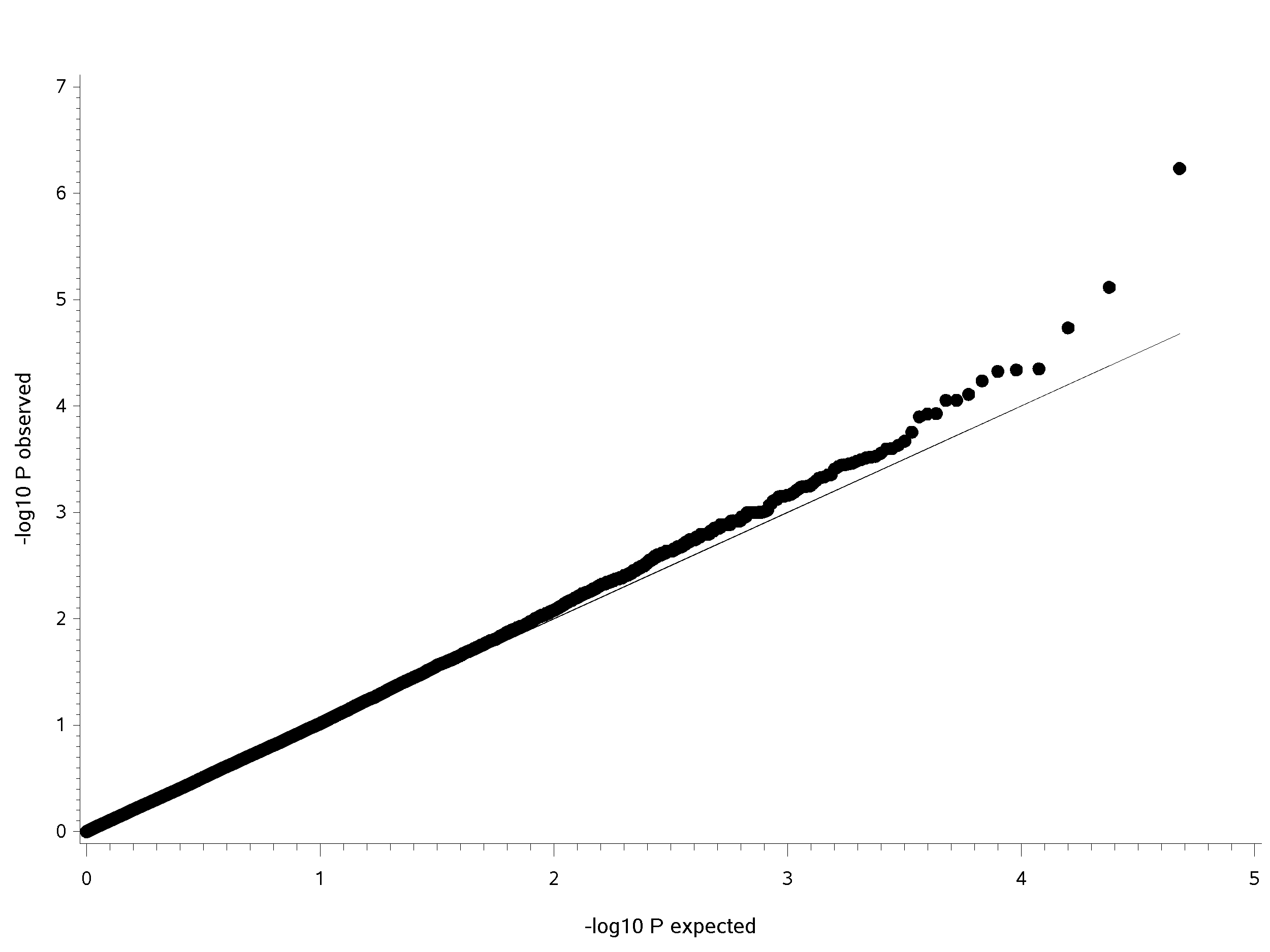


**Figure S1.** **Q-Q-plot of expected –log_10_P-values versus observed–log_10_P-values from the mixed model analysis for canine hip dysplasia score in German shepherd dogs.** Shown are all 47,729 SNPs included in the genome-wide association analysis with the grey line corresponding to the null hypothesis of no association.
