# Supplementary material for: Identification and Validation of Quantitative Trait Loci (QTL) for Canine Hip Dysplasia (CHD) in German Shepherd Dogs
Source: PLoS One. 2014 May 6;9(5):e96618. doi: 10.1371/journal.pone.0096618 (PMC4011879; doi:10.1371/journal.pone.0096618)
Supplement: Table S1 — Results of the genome-wide association study for canine hip dysplasia in 192 German Shepherd Dogs. Given are the SNP-IDs with their locations on the dog chromosome according to CanFam2.0, the minor allele frequencies (MAF) in the sample and –log10P-values (P-MLM) from the mixed linear model analysis. The SNP BICF2S2367279 exceeds the threshold (5.98) for genome-wide significance. (DOC) [file pone.0096618.s002.doc]

**Table S1.** Results of the genome-wide association study for canine hip dysplasia in 192 German Shepherd Dogs. Given are the SNP-IDs with their locations on the dog chromosome according to CanFam2.0, the minor allele frequencies (MAF) in the sample and –log_10_P-values (P-MLM) from the mixed linear model analysis. The SNP BICF2S2367279 exceeds the threshold (5.98) for genome-wide significance.

| Chromosome | SNP-ID | Position on CanFam2.0 in base pairs | MAF | P-MLM |
| --- | --- | --- | --- | --- |
| 19 | TIGRP2P265674 | 35,533,136 | 0.48 | 5.12 |
| 24 | BICF2S2367279 | 28,944,481 | 0.47 | 6.23 |
| 26 | BICF2P281364 | 17,181,662 | 0.49 | 4.35 |
| 34 | BICF2P1086886 | 4,239,914 | 0.35 | 4.73 |
| 34 | BICF2P355865 | 39,346,805 | 0.36 | 4.31 |
