# Supplementary material for: Identification and Validation of Quantitative Trait Loci (QTL) for Canine Hip Dysplasia (CHD) in German Shepherd Dogs
Source: PLoS One. 2014 May 6;9(5):e96618. doi: 10.1371/journal.pone.0096618 (PMC4011879; doi:10.1371/journal.pone.0096618)
Supplement: Table S2 — Single nucleotide polymorphisms (SNPs) with their chromosomal positions on CanFam2.0, adjacent genes, SBE primers, SBE orientations and SNP motifs genotyped in the validation set including 843 German Shepherd Dogs. (DOC) [file pone.0096618.s003.doc]

**Table S2.** Single nucleotide polymorphisms (SNPs) with their chromosomal positions on CanFam2.0, adjacent genes, SBE primers, SBE orientations and SNP motifs genotyped in the validation set including 843 German Shepherd Dogs.

| CFA | SNP  Gene | Chromosomal position (Mb) | SBE primer (5’ - 3’)  SBE orientation  SNP motif edited |
| --- | --- | --- | --- |
| 19 | TIGRP2P265674  (*DDX18*) intergenic (*DPP10*) | 35.533 | ACCAGCAGTATTAGGAAGAGATACTTG  G/A-reverse  C>T |
| 24 | BICF2S2367279  (*MANBAL*) intergenic (*SRC*) | 28.944 | GGAAGGACCTGCTGGG  G/C-reverse  C>G |
| 26 | BICF2P281364  *KSR2* (intron 5) | 17.182 | TGCTGAAGCTGTGTCCCT  T/C-reverse  A>G |
| 34 | BICF2P1086886  (*DNAH5*) intergenic (*CTNND2*) | 4.230 | CTAAAGTTAAATTAGCTAGCGGT  C/T-forward  C>T |
| 34 | BICF2P355865  (*TMEM212*) intergenic (*FNDC3B*) | 39.347 | TTGGGTCGTGTGAGGGTAG  A/G-forward  A>G |
