# Supplementary material for: Identification and Validation of Quantitative Trait Loci (QTL) for Canine Hip Dysplasia (CHD) in German Shepherd Dogs
Source: PLoS One. 2014 May 6;9(5):e96618. doi: 10.1371/journal.pone.0096618 (PMC4011879; doi:10.1371/journal.pone.0096618)
Supplement: Table S3 — Comparison of -log10P-values using a cumulative logit and a linear model for an association analysis of canine hip dysplasia genotyped in the detection sample including 192 German Shepherd Dogs considering 96 CHD-A dogs as controls and 65 mildly, 22 moderately and 9 severely CHD-affected dogs. Shown are the raw -log10P-values of the five highest CHD-associated SNPs for a model with genotype as the sole effect and the overestimation of the raw -log10P-values through a linear model. (DOC) [file pone.0096618.s004.doc]

**Table S3.** Comparison of -log_10_P-values using a cumulative logit and a linear model for an association analysis of canine hip dysplasia genotyped in the detection sample including 192 German Shepherd Dogs considering 96 CHD-A dogs as controls and 65 mildly, 22 moderately and 9 severely CHD-affected dogs. Shown are the raw -log_10_P-values of the five highest CHD-associated SNPs for a model with genotype as the sole effect and the overestimation of the raw -log_10_P-values through a linear model.

| SNP | -log_10_P-value (cumulative logit) | -log_10_P-values (linear quasi-continuous) | Estimated overestimation (%) |
| --- | --- | --- | --- |
| TIGRP2P265674 | 7.07466 | 7.10373 | 4.09 |
| BICF2S2367279 | 9.04418 | 9.35024 | 3.27 |
| BICF2P281364 | 8.03228 | 8.38242 | 4.18 |
| BICF2P1086886 | 5.10513 | 5.81499 | 12.21 |
| BICF2P355865 | 0.92070 | 1.15490 | 20.28 |
